# Antibiotics: practice and opinions of Cambodian commercial farmers, animal feed retailers and veterinarians

**DOI:** 10.1186/s13756-016-0147-y

**Published:** 2016-11-11

**Authors:** Chhorvoin Om, Mary-Louise McLaws

**Affiliations:** School of Public Health and Community Medicine, UNSW Medicine, UNSW, Level 3 Samuels Building, Sydney, 2052 NSW Australia

**Keywords:** Food animals, Feed booster, Antibiotics, Prevention, Growth promotion, Farming

## Abstract

**Background:**

Cambodia has reported multidrug resistant bacteria in poultry, similar to other countries in the region. We visited commercial food animal farms to explore opinions and antibiotic practices on the farms.

**Methods:**

We used individual in-depth qualitative interviews with 16 commercial farmers, four feed retailers and nine veterinarians from food animal industry and government offices from the southwestern region of Phnom Penh. Transcribed interviews were thematically analysed.

**Results:**

Widespread antibiotic use occurred on all farms and was driven by four facilitators: belief that antibiotics were necessary for animal raising, limited knowledge, unrestricted antibiotic access, and weak monitoring and control systems. *“If we treat ducks for two days and they aren’t cured we change to human drugs. We cocktail 10 tablets of this, 10 tablets of that and 20 tablets of this one. Altogether 200 tablets are mixed in 100 or 200 L of water for the ducks to drink. No one taught me, just my experiences.”* Antibiotics were believed to be necessary for disease prevention. *“On the first day when we bring in the chicks, we let them drink Enro [enrofloxacin] and vitamins to make them resist to the weather. We place them in the house and there are some bacteria in the environment. When they are newly arrived, we have to give them feed. So we’re afraid they get diarrhea when they eat feed, we have to use Enro.”* All farmers used pre-mixed feed that veterinarians and feed retailers acknowledged contained antibiotics but not all listed the antibiotics. Farmers viewed pre-mixed feed as a necessary ‘feed supplement’ for growth promotion. *“….The fatten supplement is mixed in feed. Pigs aren’t growing well unless I use the supplement.”* Farmers and veterinarians were concerned that ‘antibiotic residuals’ in animal meat could harm human health. But they did not link this with antibiotic resistance.

**Conclusions:**

Antibiotic use in food animals was widespread and uncontrolled. Farmers focused on the benefits of food animal production rather than concerns about the consequences of antibiotic use. Therefore, education for prudent use of antibiotics in food animals and regulations are urgently needed in food animal farming in Cambodia.

## Background

The use of antibiotics is associated with the development of antibiotic resistance [1–4] and this association has been observed since the time penicillin was introduced into clinical treatment [5, 6]. As early as 1940 Abraham et al. reported the ability of *Staphylococcus aureus* to adapt to higher concentrations of penicillin [5]. In just five years after the first use of penicillin to treat *Staphylococcus aureus* infections the level of resistance rose from less than 1 % [7] to almost 60 % [8]. The most common mechanism for bacteria to develop antibiotic resistance is through plasmid mediation [9–11] where antibiotic resistance genes can be transferred within and between bacterial species [7, 11, 12].

Food animals are routinely exposed to sub-therapeutic doses of antibiotics for the purpose of growth promotion and prophylaxis [13] and this practice commenced in high density food animal farming as early as the 1950s [13–15]. This sub-therapeutic antibiotic use in food animals has been linked to antibiotic resistance in bacteria found in food animal products and their environment [16–19]. These resistant bacteria are transmitted to humans through food consumption and direct contact with animals and their environment [13, 17, 20]. For instance, the sub-therapeutic use of enrofloxacin has been associated with increased quinolone resistance in *E. coli* isolates in poultry products [21]. Environmental spread of antibiotic resistant bacteria from swine facilities were detected in downstream water that contained antibiotic resistant enterococci and resistant *E. coli* at a concentration of up to 33 times higher than the concentration in the upstream water [22]. Residents near turkey farms where avoparcin was used for growth promotion were found to harbor vancomycin-resistant enterococci suggesting environmental transmission [23]. An example of direct transmission of resistance was reported in a study that identified methicillin resistant *Staphylococcus aureus* (MRSA) ST398 in both farmers and their broiler chickens [24].

In 2006 the European Union withdrew support for antibiotic use as a growth promoter [25]. Yet, antibiotic use in food animals, especially for growth promotion, has increased globally with the increase in food animal farming in developing countries [26]. In 2011 close to half of all the global antibiotics used in food animals was used in the Asia-Pacific region and *Salmonella*, *Campylobacter* and *E. coli* in livestock in this region were found to be multidrug resistant [27]. A study from Thailand reported multidrug resistant strains of *Salmonella enterica* isolates from poultry and swine [28]. Similarly, in Laos a study reported 73 % of *Salmonella* isolates from beef, pork and buffalo meat were multidrug resistant [29]. Multidrug resistance was also reported in *Salmonella* serovars and *Campylobacter* spp. isolates from poultry meat in the Cambodian capital, Phnom Penh [30]. Many studies have identified pathogenic bacteria and multidrug resistance in food animals but omit exploring farmers’ practices and opinions about antibiotic use. We went to farms in the southwestern region of the Cambodian capital city, Phnom Penh, to explore practices and opinions of commercial farmers in association with antibiotic use and obtained confirmation from feed retailers and veterinarians.

## Methods

### Study design and setting

This qualitative study used individual in-depth interviews between December 2013 and February 2014. Cambodia is a low-income country with a population of 15.33 million [31] who predominantly reside in rural areas [31, 32]. Traditionally, Cambodian households raise poultry and pigs in their backyards for food and to supplement their household income [33–36]. The number of backyard animals varied from one to six pigs [36], five to 13 ducks and 18 to 37 chickens [34]. Commercial poultry production supported by foreign investment commenced in Cambodia in mid-1990s [34]. The average number of animals on commercial poultry farms is 3500 broiler chickens, 5000 layer chickens and 900 ducks [34]. The pig population for the whole country has been estimated at about 2 million with 80 % raised in household backyards [36, 37]. There are only three commercial industries that raise pigs and supply grandparent stock, breeding sows, piglets, feeds and veterinary products to farmers [36]. Commercial pig farmers raise between 10 and 30 pigs, normally feed their animals a mixture of rice, commercial food and vegetables while “some farmers” [36] were reported to also use medicated feed or growth promoting feed [36]. Feed and veterinary products including antibiotics for pigs and poultry are locally produced, imported and distributed by various private local and foreign companies [34, 36]. Three categories of commercial food animal farming occurs in Cambodia: contracted, semi-contracted and non-contracted farms [34]. Contracted farmers provide labor and animal housing while the contracting industry provides the animals, feed, veterinary products, and technical supports to raise the animals. Contracted farmers are remunerated according to the number of animals they produce. Semi-contracted farmers sell the food animals to the contracting industry at an agreed price while these farmers provide their own animals, animal feed, and veterinary products. Semi-contracted farmers receive limited technical advice from the contracting industry. Non-contracted farmers are independent of the contracting industry.

### Sampling, data collection and analysis

Participants included broiler poultry and pig farmers, animal feed retailers, food animal industry veterinarians (industry veterinarians), and governmental veterinarians. Participants were recruited using purposive sampling [38]. We held interviews with farmers on their farms and also made field observations. Feed retailers were interviewed in their shop and veterinarians were interviewed in their office. Farmers were from the southwest of Phnom Penh, Cambodia, because farming was concentrated in this region. Individual interviews continued until data saturation when no new information was elicited [39, 40]. Photographs of feed bags and antibiotics used on the farm were taken with permission. A Khmer speaking physician (CO) conducted all interviews. All interviews were digitally recorded and transcribed verbatim by an experienced transcriber and an independent local medical doctor checked transcripts against digital recordings before translation into English. During data coding one author crosschecked any unclear translations against the transcribed Khmer transcripts. To improve validity, data were inductively coded by both authors and checked for consistency and any discrepancies were discussed. Thematic data analysis technique was used to guide analysis and the results were presented as thematic syntheses [41, 42]. Nvivo version 10 was used to facilitate data coding and analysis. Field notes were also used to assist interpretation.

### Ethical consideration

The study was approved by the Cambodian Department of Animal Health and Production, Ministry of Agriculture and Fishery and Forestry, and UNSW Australia. All participants signed consent form for interview.

## Results

Interviews were given on farms by 16 farmers (seven contracted, four semi-contracted and five non-contracted), nine government and industry veterinarians and four animal feed retailers. The farm size varied between 3000 and 4500 broiler chickens, between 500 and 1000 broiler ducks, between 70 and 150 pigs with the exception of one farm that had 1000 pigs. Antibiotics were widely used on all farms participating in this study and all interviews and observations indicated that antibiotic practices were uncontrolled. We report factors that contributed to antibiotic use and concerns that were raised by participants about antibiotic use in food animals.

### Antibiotics necessary for food animal production

Interviews with farmers, veterinarians and animal feed retailers and field observations identified a broad range of antibiotics used on food animal farms including beta-lactam, fluoroquinolones, tetracycline, colistin and lincosamides. Farmers and veterinarians commonly spoke of ‘antibiotics’ being useful for the prevention and treatment of diseases in their animals and believed that without antibiotics, their livestock would not thrive. All poultry and pig farmers administered antibiotics in the water for disease prevention and treatment. For treatment and prevention of diarrhea and respiratory infections with antibiotics known by name, all farmers reported having used enrofloxacin and amoxicillin. During field visits to poultry farms all used a packaged amoxicillin-colistin mixture for respiratory and gastrointestinal infections.

**Semi-contracted chicken farmer (** ***140120_002*** **):** *If we don’t use any antibiotics at all, when the chicken get sick the chicken will not recover.*

**Contracted chicken farmer (** ***131208_005*** **)**: *If it was severe diarrhea, we give Amox [amoxicillin] to stop it. Amox can help heal the colon and stomach of the chicken so that they do not get much diarrhea.*

**Non-contracted duck farmer (** ***140120_005*** **):** *This one is Enro [enrofloxacin] for preventing diarrhea when the ducks are small. It is good to use [enrofloxacin] one or two courses.*

**Non-contracted pig farmer (** ***140206_001*** **):** *When pigs get sick for example if they have diarrhea, I would buy an injectable antibiotic, Enro [enrofloxacin] [but] it depends on the type of disease, I use injections when pigs have diarrhea or fever. If the pigs dislocates its joint leg, I would give them Amox [amoxicilin] and Dexa [Dexamethasone] injection. I didn’t study anything but I just learnt it from the vet I often called to give injections to my pigs in the past.*

Industry veterinarians who advised on a daily basis contracted chicken farmers described the routine use of antibiotics:

**Industry veterinarian (** ***13219_003*** **)**: *On the first day when we bring in the chicks, we let them drink Enro [enrofloxacin] and vitamin in order to make them resist to the weather. We place them in the house and there are some bacteria in the environment. When they newly arrive, we have to give them feed. So we are afraid they get diarrhea when they eat feed, we have to use Enro.*

**Industry veterinarian (** ***131224_002*** **)**: *If we talk about chicken raisers, all of them use antibiotics. No-one doesn’t use them. Farmers want chickens with meat. If chickens eat feed and get diarrhea, the chickens will not have meat. Antibiotics reduce diseases and provide quality meat.*

Although farmers and veterinarians did not explicitly use the term ‘growth promotion’ their purpose of antibiotic use was to promote growth. They commonly spoke about a belief that antibiotics facilitated their animals’ ability to ‘fight off diseases’, given as the common cold and diarrhea, that would reduce meat production. Based on observations and discussions with veterinarians antibiotics were always present in the feed products for pigs. A pig famer used feed mixture containing doxycycline and tylosine, or chlortetracycline while others used feed labelled as containing ‘antibiotics’ but these were not specified. Farmers referred to these types of feed as ‘feed booster’, ‘feed master’ or ‘feed supplement’ and used these to boost growth.

**Non-contracted pig farmer (131208_002):** *I buy fatten supplement from company. The fatten supplement is mixed in feed. Pigs are not growing well unless I use the supplement.*

**Non-contracted pig farmer (** ***140206_001*** **):** *I bought feed booster [with doxycycline and tylosine] to mix with husk and rice distilled from alcohol. After weaning we mixed it for the piglets to eat a little to fight off diseases. It is effective and the pigs will eat more. They don’t get sick, no diarrhea. It’s for all pigs, piglets and adult pigs.*

Observations of poultry animal feed bags were labelled using a code or labelled as ‘feed additive’. Of the farming industry and government veterinarians and feed retailers who were interviewed all reported that these additives included antibiotics.

**Senior government veterinarian (131217_001)**: *Locally produced feed is mixed in with antibiotics but the thing is that we don’t know how much antibiotics are mixed. They include some substances and antibiotics to fight off diseases like diarrhea.*

**Feed retailer (** ***131208_007*** **):** *There might be antibiotics in it [the feed], there might be supplements of growth hormones or there could be chemical substances which we don’t know about. But I think there is an addictive substance included so when the animal eats it they become addicted and grow fat.*

### Limited understanding of antibiotics

Knowledge of antibiotics among farmers was limited to knowing the names of some antibiotics they used routinely, such as enrofloxacin and amoxicillin. None of the 16 farmers exhibited an understanding about the actions and indications for antibiotics. They explained that they relied on the logo and color of the label to identify the antibiotics for the treatment of sick animals and choice and dosage were not pharmacologically based, only experiential. Non-contracted broiler duck farmers admitted to treating their animals with human antibiotics.

**Semi-contracted chicken farmer (** ***131208_005*** **):** *This is a diarrhea medicine. It wasn’t effective. It only worked when I used the blue one instead. I don’t know the name of the medicine. They [industry] wouldn’t tell me their most effective medicine.*

**Fig. 1:**
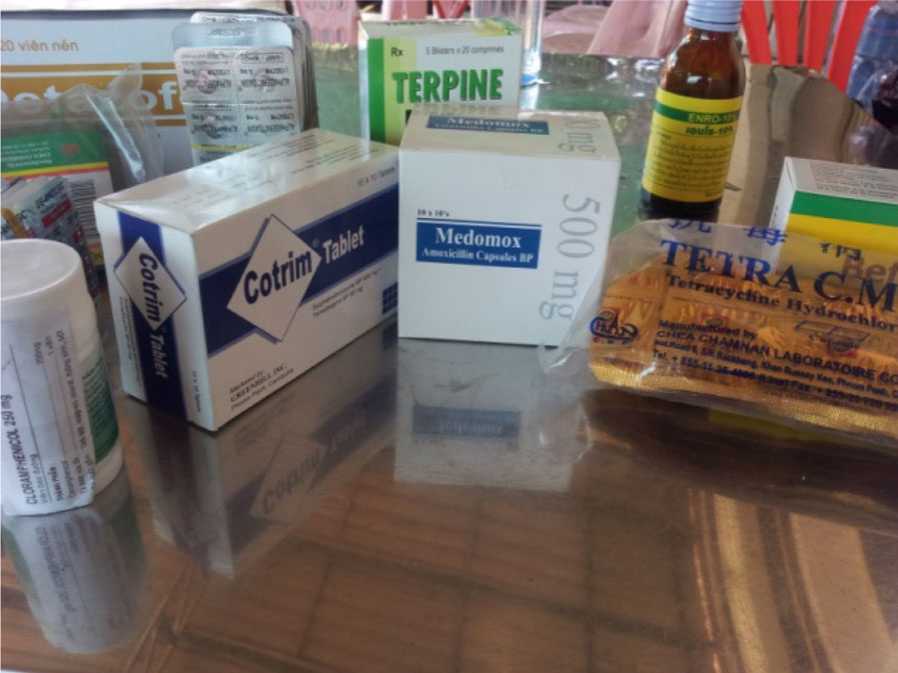
Human medicines used to make a cocktail for the treatment of broiler ducks

**Non-contracted duck farmer (** ***131207_001*** **):** *If we treat ducks for two days and they aren’t cured we change to human drugs. We cocktail ‘10 tablets of this’ [points to drugs] ‘10 tablets of that’ [points to another drug] and ‘20 tablets of this one’ [points to another drug]. Altogether 200 tablets are mixed in 100 or 200 l of water for the ducks to drink. No one taught me, just my experiences* (See Fig. 1).

Semi-contracted and non-contracted farmers described unconventional treatment regimens while contracted-farmers understood that the contract industry veterinarian was responsible for treatment.

**Semi-contracted chicken farmer (** ***140120_002*** **):** *Antibiotics are normally used for treatment. Sometimes the chickens are not fine and we cannot find the causes. So it’s good to treat them ‘bit by bit’ so that it won’t get serious, and the vet also recommended me to use like that.*

**Contracted chicken farmer (** ***131208_001*** **):** *When the chickens have bloody diarrhea, we see the stool and we see bloody diarrhea and so on, we notify the company’s vet to come. The vet would bring the medicine for us to mix with water for the animals to drink and so on.*

Imported antibiotics were not labeled in Khmer. This challenged not just the farmers but also veterinarians and animal feed retailers who give advice to farmers.

**Non-contracted duck farmer (** ***140120_005*** **)** *: I can’t read, I just tell them [retailers] I want to buy medicine with the logo of a chicken with its head tied up.*

**Contracted chicken farmer** ***(131208_005*** **):** *It’s a diarrhea medicine. It was not effective only when I turn to use the blue one instead. I don’t know the name of those medicines.*

**Contracted chicken farmer (** ***131208_001*** **):** *I don’t know the name of the medicines. There are types of antibiotics. All are written in English and I don’t know English.*

**Feed retailer (** ***131208_008*** **):** *Yes, if we buy products which don’t have Khmer written on them, it’s difficult. I want all imported medical products to Cambodia to have Khmer written on them so that the farmers find it easy to read. Not just farmers, even me who as a seller, without Khmer letters written on them, I also find it hard to read.*

### Unrestricted access to veterinary antibiotics

Antibiotics can be purchased without veterinary prescription from any animal feed retailers. Although all retailers may prescribe animal antibiotics not all were trained veterinarians.

**Semi-contracted pig farmer (131208_002):** *I buy the drugs directly from drug store by telling them the symptoms. They’ll provide the drugs according to the symptoms. Sometimes, there are 2 to 3 types of drugs which I can’t remember the name because I can’t read the foreign letters.*

**Feed supplier (131208_007):** *For example, this month most animals had a bad cold and red spots on their bodies. I cocktailed drugs for them, for the red spots I would cocktail antibiotic like linco [lincomycin] plus a cooling [antipyretic] medicine*.

There are private companies outside the contracting industry that produce and import feed and antibiotics. These companies market antibiotics and veterinary products directly to semi- and non-contracted farmers. These companies explain to farmers the use of their products or they invite farmers to participate in training sessions on how to use their products. Contracting industries also promote their products to their farmers.

**Industry veterinarian (131224_002):** *They [farmers] can ask [private] companies to come to their farms. These companies instruct them on the use of lots of antibiotics, almost all types*.

**Senior government veterinarian (131217_001):** *Companies advertise to compete with each other by saying that the products from their companies are much better, make animals healthy because the feeds have enough proteins and so on, and the animals would not get sick.*

**Semi-contracted pig farmer (** ***140120_003*** **)**: *The [contracting] company invited me to join their training. For instance, the company has medicines and they want to sell them, so, they called me to join the training to know how to use them.*

### A weak monitoring and control system

The Cambodian government veterinarian advisory system works at central level, provincial, district and village level. Experienced farmers are trained in basic animal husbandry to become part of the veterinarian advisory system at the village level. These advisory farmers prescribe antibiotics for household food animals but are not used by their peer non-contracted farmers as a source of information or treatment. There are other factors that adversely impact the capacity to control antibiotic use in food animals. These include limited expertise in staff from the agricultural departments to monitor farming practices and limited financial and material support to test for antibiotic resistance. The activities of the governmental veterinarians focused on household pigs and cows but not commercial farms. The management of commercial food animals was through industry and the individual farmer.

**Senior government veterinarian (131217_001):** *How many specialists do we have so far at the animal service department [not many]? Another thing is material, is our facility sufficiently equipped? And can we detect problems yet? Another problem is budget. If you ask why the budget is relevant because when you go to field to do research, we need money.*

**District government veterinarian (140206_004):** *For chicken and ducks we don’t focus much on them. We [government services] focus heavily only on preventive and treatment measures on cows and pigs.*

There is no control of antibiotic use by non-contracted and semi-contracted farmers. Although contracted farmers use antibiotics provided by the contracting industry our observations and discussions revealed that there was no guarantee that antibiotic use by contracted farmers was appropriately controlled.

**Industry veterinarian (131224_002):** *No-one controls these [semi- and non-contracted] sectors. For the company, this isn’t a problem. In feed, there could be a mixture of some [antibiotics] but by the age of 35 days chickens are not allow to eat feeds with antibiotics. So, there is a window of 10 days that chickens should eat feed without any drugs before being slaughtered.*

### Concerns about antibiotic use

Despite focusing on the benefits of antibiotic use on production participants expressed concerns about potential consequences of antibiotic use in food animals on humans. Farmers, veterinarians and animal feed retailers spoke about their concerns for ‘antibiotic residuals’ in the meat and adverse impacts on the growth of animals.

**Non-contracted pig farmer (140206_001):** *I think antibiotics will have consequences on people who eat the meat. That’s why it is good if we keep those pigs for one week or so without antibiotics before we can sell them.*

**Industry veterinarian (131224_002):** *Based on my limited knowledge there’s a possible impact. When we use amoxicillin in animals the residual may remain in the animal when it’s sent to slaughter. If we eat this meat it will impact our health. When we use this kind of antibiotic on ourselves it will not be effective because of the residual [resistance] in the food.*

A fear of the consequences from antibiotic use in their animals was raised during interviews. Farmers and feed retailers spoke about their experience of animals dying when they received an ‘overdose’ of antibiotics and about their animals stopped growing when they consume ‘too much’ antibiotics.

**Non-contracted pig famer (140206_001)** ***:*** *When we overdose* [the animals] *it will have consequences in the pigs. It can make the pigs shake and stop growing.*

**Non-contracted chicken farmer (131207_002):** *Actually I need to limit* [antibiotics]*. I don’t want to use it much because using medicines can lead to the reduction of our yields.*

**Contracted chicken farmer (131208_006)** ***:*** *If we use amox* [amoxicillin] *a lot the chicken won’t grow. When the chicken gets recovered from the sickness they still won’t grow up.*

**Animal feed supplier (131208_007):** *The consequence is not so huge because we don’t use them* [antibiotics] *much. Yes, but antibiotics really can make the pigs stop growing.*

When the interviewer raised the possibility that humans who consume meat from antibiotic-fed animals may produce antibiotic resistance in the human, none of the farmers had heard of this link. However, a veterinarian expressed a concern related to such a link.

**Senior government veterinarian (131217_001):** *When we eat the meat, usually having drug substances, it’s no different from taking a medicine, limited dose, low dose. It’s not right. We absorb antibiotics too. They act by telling the microorganisms inside our bodies that there is antibiotic coming. Day one, microorganisms are in coma. Day two, they are better and on day three, these microorganisms are fine, no problem. It is like that.*

## Discussion

We went to commercial food animal farms and found antibiotics were used widely and uncontrolled on all participating farms. Farms listed in the region from which we recruited included 69 commercial broiler chicken farms (mostly contracted) and 10 broiler duck farms (unknown contract category) but there were no data on commercial pig farms [34]. The farming industry, feed retailers and veterinarians associated with our 16 farms also have dealings with farms across this region. The marketing of products by industry will no doubt be similar across the region and we therefore believe the practices we have reported here are common to other farms. These practices were guided by the commercial farming industry and feed retailers. The labelling of pre-mixed feeds containing antibiotics did not always specify the antibiotic by name and labels were not always in Khmer. These two practices prevented farmers from making an informed decision about their antibiotic use. Access to antibiotics was not restricted as farmers could purchase any drug from retailers without prescription. These findings suggest that measures to ensure prudent use of antibiotics by food animal farmers and monitoring programs were lacking and our findings concur with a seminal review of practices in eight Southeast Asian countries [43].

An antibiotic-free period in the animals before slaughter could not be evaluated on any of the farms for us to establish whether these practices correspond with international guidelines [44]. Of major concern was the practice of using human antibiotics as the last resort to treat animals not responding to drug cocktails.

The majority of *Salmonella* and *Campylobacter* species sampled from chicken meat retailers in Phnom Penh markets were resistant to nalidixic acid, amoxicillin, ciprofloxacin and cefalotin [30]. Based on these findings [30] and our observations of widespread unrestricted antibiotic use we speculate that there is a relationship between antibiotic use on the farms and resistance in the food animal meat samples. This relationship was found elsewhere [45, 46]. Vancomycin resistant *E. faecalis* isolates from food animals were associated only on farms where avoparcin had been used [45]. The use of fluoroquinolones, such as enrofloxacin, in poultry was associated with ciprofloxacin-resistant *Campylobacter jejuni* isolates from both poultry and human [46] resulting in the withdrawal of fluoroquinolones from poultry farming in 2005 in the United States [47].

Recent microbiological studies in Cambodia reported a significant increase in the resistance to ciprofloxacin in *Salmonella typhi* isolates from human bloodstream infections [48–50]. Although there is an absence of a definitive study examining the association between such resistance in both humans and food animals, we are particularly concerned because (i) enrofloxacin was widely used in all participating food animal farms (ii) resistant genes can be transferred within and between bacterial species [51, 52] and (iii) bacteria resistant to one class of antibiotic administered to food animals are capable of developing resistance to antibiotics of similar classes administered to humans [13].

We cannot speculate on the antibiotic misuse in food animals kept in backyards in Cambodia. However, based on other studies into environmental contamination of water sources [22, 23] we believe there is a potential for the spread of antibiotic resistant bacteria to both residents and animals located around our participating farms.

Our farmers could not express directly their concerns about adverse effects of antibiotics in terms of resistance. But they did express concerns about adverse effects in humans from ‘antibiotic residuals’ in animals prior to being slaughtered and eaten. These concerns about the ‘antibiotic residual’ effects in the meat could be utilized by veterinarians and government agencies to educate farmers to reduce indiscriminate antibiotic use. Education of farmers should not be provided by the industry which may be self-serving. However, as antibiotics are mostly administered in pre-mixed feed the reduction of antibiotics would have to start with policies directed at the contracting industry and feed retailers who supply feed as these represent the entire source of feeds to farmers. Weak capacity of monitoring animal husbandry and control of antibiotic use in livestock is similar in some countries in the region [43].

## Conclusions

The antibiotic practices in food animals were inappropriate and uncontrolled. Farmers focused on production benefits from antibiotics rather than the consequences of antibiotic use. Regulation, monitoring and control program for prudent use of antibiotics in food animals must begin with feed retailers and commercial industry. Labelling of all veterinary products must be in Khmer. Prudent use of antibiotics must begin with knowledgeable farmers and their training should be provided by an agency independent of the industry.

## Abbreviations

UNSW: University of New South Wales
WHO: World Health Organization
